# Efficacy and Safety of Transcranial Direct Current Stimulation as an Add‐On Trial Treatment for Acute Bipolar Depression Patients With Suicidal Ideation

**DOI:** 10.1111/cns.70077

**Published:** 2024-10-09

**Authors:** Dandan Wang, Xiaonan Guo, Qi Huang, Zhong Wang, Jingkai Chen, Shaohua Hu

**Affiliations:** ^1^ Department of Psychiatry, The First Affiliated Hospital Zhejiang University School of Medicine Hangzhou China; ^2^ The Zhejiang Key Laboratory of Precision Psychiatry Hangzhou China; ^3^ Nanchong Psychosomatic Hospital Nanchong China; ^4^ Nanhu Brain‐computer Interface Institute Hangzhou China; ^5^ Zhejiang Engineering Center for Mathematical Mental Health Hangzhou China; ^6^ School of Brain Science and Brian Medicine, and MOE Frontier Science Center for Brain Science and Brain‐Machine Integration Zhejiang University School of Medicine Hangzhou China

**Keywords:** bipolar disorder, depression, dorsal lateral prefrontal cortex, suicidal ideation, transcranial direct current stimulation

## Abstract

**Aims:**

Bipolar depression poses an overwhelming suicide risk. We aimed to examine the efficacy and safety of transcranial direct current stimulation (tDCS) combined with quetiapine in bipolar patients as a suicidal intervention.

**Methods:**

In a single‐center, double‐blind, treatment‐naive bipolar depression patients with suicidal ideation were randomly assigned to quetiapine in combination with either active (*n* = 16) or sham (*n* = 15) tDCS over the left dorsolateral prefrontal cortex for three consecutive weeks. The 30‐min, 2‐mA tDCS was conducted twice a day on the weekday of the first week and then once a day on the weekdays of the two following weeks. Primary efficacy outcome measure was the change in the Beck Scale for Suicidal Ideation (BSSI). Secondary outcomes included changes on the 17‐item Hamilton Depression Rating Scale (HDRS‐17) and Montgomery‐Asberg Depression Rating Scale (MADRS). Outcome was evaluated on Day 3 and weekend. Safety outcome was based on the reported adverse reactions.

**Results:**

Active tDCS was superior to sham tDCS on the BSSI at Day 3 and tended to sustain every weekend during the treatment process, compared to baseline. However, no difference between active and sham in HDRS‐17 and MADRS was found. Response and remission rate also supported the antisuicide effect of tDCS, with higher response and remission rate in BSSI, but no antidepressant effect, compared to sham, over time. Regarding safety, active tDCS was well tolerated and all the adverse reactions reported were mild and limited to transient scalp discomfort.

**Conclusion:**

The tDCS was effective as an antisuicide treatment for acute bipolar depression patients with suicidal ideation, with minimal side effects reported.

## Graphical abstract

Active tDCS was superior than sham control in decreasing BSSI score, and this rapid antisuicide effect of active tDCS was revealed within 3‐day time window.

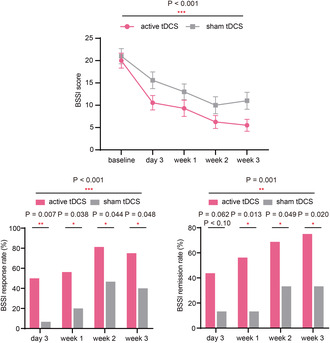

**Abbreviations**
BSSI: Beck Scale for Suicidal Ideation
DLPFC: dorsal lateral prefrontal cortex
HDRS‐17: 17‐item Hamilton Depression Rating Scale
MADRS: Montgomery‐Asberg Depression Rating Scale
tDCS: transcranial direct current stimulation

## 1 Introduction

Bipolar disorder (BD) is a debilitating mood disorder characterized by mood and energy fluctuation [1]. The lifetime prevalence of bipolar spectrum disorder is as high as 2.4%, bringing a giant burden for society [2, 3]. Strikingly, it was confirmed that the suicide risk of BD patients is about 20–30 times higher than the general population, representing the highest risk of successful suicide and the most frequent suicidal behavior among all mental disorders [4, 5, 6, 7, 8]. Therefore, acute suicide intervention for BD patients with suicidal ideation is under urgent demand.

Currently, the main interventions for BD patients with suicide ideation can be divided into drug therapy and nondrug therapy. Though drugs like lithium, antidepressants, antipsychotics, and clozapine are approved for the treatment of suicidal behavior, they have their own limitations including huge side effects and contradictory outcomes [9]. For instance, lithium is still controversial for its antisuicide effects. While some results proved that the suicide risk of BD patients after lithium treatment was significantly reduced [10], others reported that lithium had no significant antisuicide effect [11]. For nonpharmacological treatment, electroconvulsive therapy (ECT) is proven to function. However, due to the intolerance of side effects, the clinical application of ECR is still far more limited despite a good effect on suicide risk prevention [12].

Instead of systematic treatment, recent advances have also shed light on the antisuicide effect of novel noninvasive neuromodulation techniques, such as transcranial magnetic stimulation (rTMS) and transcranial direct current stimulation (tDCS). rTMS employs electromagnetic pulses to induce electric currents in the brain, targeting specific cortical regions. It is typically administered using a figure‐of‐eight coil placed over the scalp, allowing for deeper penetration and more focal stimulation. In contrast, rtDCS involves the application of weak direct currents through electrodes placed on the scalp, resulting in a more diffuse modulation of cortical excitability [13]. The dorsolateral prefrontal cortex (DLPFC), acting as a cognitive and emotion regulation center, wins the most attention in suicidal ideation of depression patients [14, 15, 16]. Weissman et al. [17] and Pan et al. [18] showed that active rTMS over DLPFC was more effective than sham rTMS stimulation in reducing suicidal ideation of unipolar depression patients, indicating DLPFC as a potential target to intervene in suicide ideation. In addition to traditional rTMS, tDCS is proposed to modulate prefrontal cortex synaptic plasticity and metabolic activity [19, 20, 21], which orchestrates the long‐term effect of symptom improvement. Clinical trials conducted with tDCS alone or with sertraline were superior to the placebo effect on diminishing the suicidal thoughts for unipolar depression patients [22, 23]. However, to date, clinical experiments employing novel noninvasive neural modulation techniques have lacked firm evidence of antisuicide efficacy in patients with bipolar depression.

Considering tDCS has numerous clinical advantages including being cheaper than the rTMS and more convenient to use at home, it will be more approachable for the patients than receiving rTMS therapy. Hence, to address the question of whether tDCS over left DLPFC is effective and safe for bipolar depression patients with suicidal ideation, we first sought to design the double‐blinded, randomized, sham‐controlled trial, hoping to provide valuable evidence for suicide intervention clinical practice in BD.

## 2 Methods

### 2.1 Participants

Participants were recruited through media advertisements and physician referrals. Eligible subjects were bipolar disorder outpatients or inpatients, aged 15–45, with a *Diagnostic and Statistical Manual of Mental Disorders, Fifth Edition* (DSM‐V) diagnosis of bipolar disorder, with a primary or recurrent depression episode. Suicidal ideation in the last week was the required feature of the patients. The required episode had a 17‐item Hamilton Depression Rating Scale (HDRS‐17) [24] total score of at least 17 and its item‐3 score of at least 3, as well as a Beck Scale for Suicidal Ideation (BSSI) [25] score of at least 12. There was no restriction in terms of sex and the first or relapsed episode. All female participants were required not to take oral contraceptives. Patients with first‐episode bipolar depression who have not taken antidepressants or mood stabilizers and patients who relapsed did not take antidepressants or mood stabilizers for at least 2 weeks were recognized as medication‐free and then recruited.

Exclusionary criteria for study participation included lifetime history of other psychiatric disorders that meet the DSM‐V diagnosis, such as schizophrenia, major depression, posttraumatic stress disorder, alcohol or drug dependence, and abuse; personality disorders; current infections, trauma, autoimmune diseases or other medical conditions; hormone therapy; craniocerebral injury or coma; seizures or epilepsy history or family history; pregnant or lactating; MRI evidence of brain structural abnormalities.

Withdrawal criteria were demonstrating withdrawn participation at request from the patients; worsened condition, such as serious clinical and psychiatric events, suicidal behaviors, or adverse events during the trial; tDCS intolerance; other serious physical diseases that occurred during the experiment; any intervention change during the study period.

In cases of possible exclusion due to safety reasons or serious clinical or psychiatric events, participants would be assessed separately by a trained psychiatrist from the hospital who was blind to the experiment design. Such cases, however, were not present during the experiment.

Given the sample size, we calculated the sample size using G*Power 3.1.9.7 software. With an effect size estimated to be 0.9, the power and α were set to 0.95 and 0.05, respectively. Considering the *t*‐test to compare the active and sham group differences in decreased scores from the baseline in BSSI, HDRS‐17, and MADRS, the minimum sample size was analyzed to be 34. In our small sample size study (active tDCS, *n* = 16; sham tDCS, *n* = 15), we did not reach the minimum numbers needed for statistical efficacy but could provide a potentially valuable efficacy trend.

### 2.2 Study Overview

The study was conducted in the Department of Psychiatry, the First Affiliated Hospital, Zhejiang University School of Medicine, with enrollment from was extracted from January 2021 to March 2023. This study was approved by the Clinical Research Ethics Committee of the First Affiliated Hospital, Zhejiang University School of Medicine (NCT0559646). All patients signed the written informed consent by themselves or their legal guardians and volunteers to participate in the study.

The study had two phases: a 1‐week acute treatment phase and a 2‐week maintenance phase. Patients were firstly randomized about 1:1 to either active tDCS or sham tDCS according to the random number table. During the acute trial, tDCS sessions were scheduled twice a day in a 5‐day sequence, for a total of 10 sessions. During the maintenance phase, tDCS sessions were scheduled daily also in a 5‐day sequence, for a total of 10 sessions. Typically, it was administered on a Monday to Friday.

### 2.3 tDCS Session Procedures and Parameters

The tDCS sessions were delivered employing the transcranial direct current stimulator VC‐8000F (Volcan, Nanjing). The procedures were performed by blinded and trained personnel. The anode and cathode electrodes were placed over the left and right DLPFC, respectively. Patients received no specific instructions during the trials.

For the active tDCS group, patients received 12 2‐mA sessions with the stimulation area around 25 cm^2^. The ramp‐up and ramp‐down periods were 10 s. For the sham tDCS group, patients received the same number of trials. Each tDCS trial also lasted 20 s (i.e., the time of introduction and withdrawal was also 10 s each, during which there was no stimulus). The treatment time arrangement was identical to the active tDCS group. The patients were not familiar with the differences between the sham tDCS and active tDCS in acoustic and tactile facets. Adverse events were recorded during and after each treatment session.

### 2.4 Containment Treatment

While receiving active or sham tDCS, all the subjects enrolled in this study were taking medication programs of quetiapine fumarate tablets (target dose 300 mg/day), which was considered first‐line pharmacotherapies per Canadian Network for Mood and Anxiety Treatments (CANMAT) guidelines for bipolar I and II depressive episodes [26].

### 2.5 Efficacy Assessment

The severity of suicidal ideation was evaluated by the BSSI at baseline, Day 3, and every weekend for a total of 3 weeks. Additionally, HDRS‐17 and the Montgomery‐Asberg Depression Rating Scale (MADRS) were adopted to measure the severity of depression at baseline, Day 3, and every weekend for a total of 3 weeks. The primary efficacy outcome measure was the change in the BSSI. Secondary outcomes included changes in HDRS‐17 and MADRS [27]. Concerning the categoric efficacy outcome, the response was defined as at least a 50% reduction from the baseline score. Remission was defined as the absolute score lower than 8 in BSSI, 8 in HDRS‐17, and 10 in MADRS. The response and remission rates were also analyzed.

### 2.6 Safety Assessment

Safety was self‐reported and measured at every trial visit by recording spontaneous adverse event reports. In particular, the mania switch was evaluated by blind clinical doctors based on The Young Mania Rating Scale (YMRS) score [28].

### 2.7 Statistical Analysis

All statistical analyses were performed with SPSS (SPSS, Chicago, IL) version 24.0 for Windows. The normality of the data was first examined with the Shapiro–Wilk test. All demographic data except sex, and clinical continuous variable, were examined with two‐sample *t*‐tests or Mann–Whitney *U* test based on the normality of the data. The sex data, response rate, and remission rate were analyzed with the Chi‐square test. The differences in changes in BSSI, HDRS‐17, and MADRS scores from baseline between the active tDCS group and the sham tDCS group were compared by the analysis of covariance with baseline data as a covariate. To examine the effect between active tDCS and sham tDCS during the whole trial, we adopted the two‐way ANOVA to compare the simple mean effect of tDCS intervention. All statistical tests were two‐tailed. The threshold of statistically significant difference was defined as *p* < 0.05.

## 3 Results

### 3.1 Patients Disposition

**FIGURE 1:**
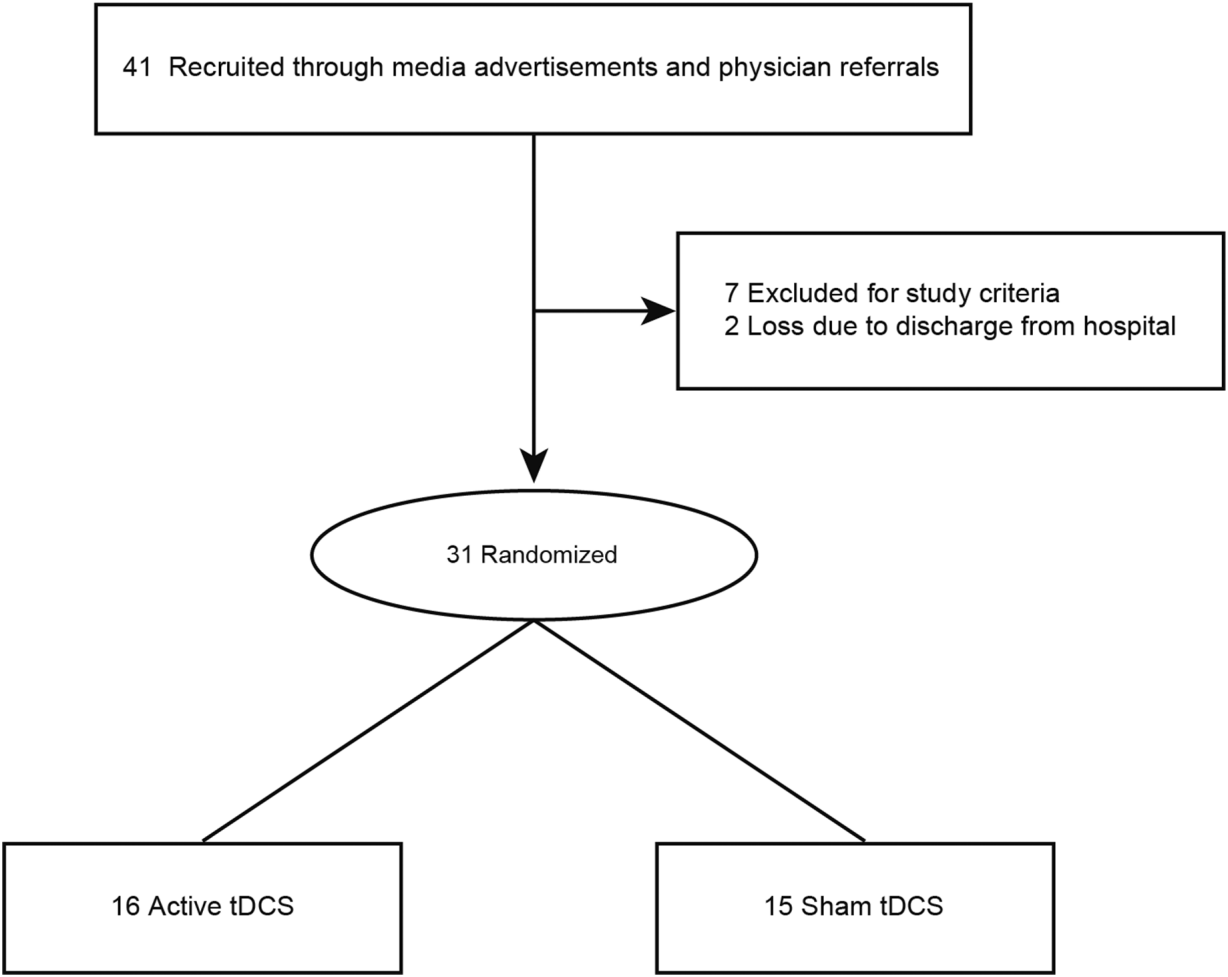
Flow diagram of participant selection.

Altogether, 40 bipolar depression patients with suicidal ideation who did not take medication within 2 weeks of the first episode or relapse were recruited, whereas seven subjects were excluded in terms of the study criteria and two withdrew from clinical assessment for discharging with symptom improvement before participating in the clinical trial. Totally, 31 subjects were randomly assigned to the active (*n* = 16) or sham (*n* = 15) tDCS and completed the final clinical measurement (Figure 1; Table 1). There were no demographic or clinical differences at baseline between the active or sham tDCS group (Table 1). The average BSSI baseline was 20.0 and 21.0 in the active or sham tDCS group, respectively.

**TABLE 1:** Demographic and clinical characteristics of all the participants.

	Active tDCS (*n* = 16)	Sham tDCS (*n* = 15)	*p*
Sex			
Male (*n*, %)	6 (37.5)	7 (46.7)	0.605
Female (*n*, %)	10 (62.5)	8 (53.3)	
Age (year)	19.38 ± 6.19	17.60 ± 2.35	0.561
Education (year)	11.63 ± 2.36	11.53 ± 1.99	0.908
Course of disease (month)	38.19 ± 19.09	35.27 ± 19.64	0.678
Baseline BSSI	20.00 ± 6.70	21.00 ± 6.57	0.678
Baseline HDRS‐17	25.25 ± 4.48	25.33 ± 7.67	0.971
Baseline MADRS	34.00 ± 5.94	33.93 ± 9.25	0.981
Baseline YMRS	2.81 ± 2.43	2.87 ± 2.50	0.952
Baseline HDRS‐17 item 3	3.19 ± 0.54	3.33 ± 0.62	0.490
Baseline MADRS item 10	4.19 ± 1.28	4.20 ± 0.86	0.975
Baseline SHAPS	35.75 ± 4.92	37.40 ± 7.25	0.462

### 3.2 Continuous Efficacy Outcome

**FIGURE 2:**
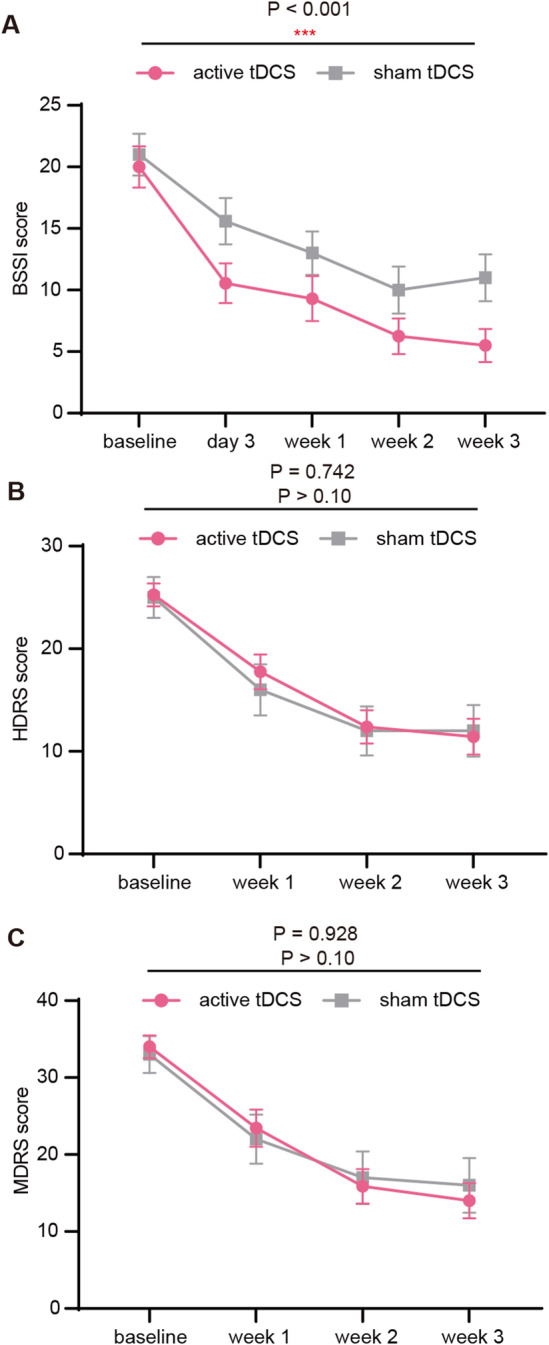
Changes in BSSI, HDRS‐17, and MDRS scores over time. (A–C) Mean changes in BSSI (A), HDRS‐17 (B), and MDRS (C) from baseline to endpoint. Active tDCS was superior to sham in BSSI score, but not in HDRS‐17 and MDRS. Error bars indicate 1 SEM.

Efficacy results for continuous outcomes with the BSSI, HDRS‐17, and MADRS are shown in Figure 2 and Table 2. BSSI decrease score at the primary efficacy time point (Day 3) was treated as the primary efficacy outcome, while the decreased score in HDRS‐17 and MADRS together with the response rate of BSSI, HDRS‐17, and MADRS were treated as secondary efficacy outcomes.

**TABLE 2:** Clinical characteristics difference after active and sham tDCS intervention.

	Active tDCS (*n* = 16)		Sham tDCS (*n* = 15)		Active vs. Sham
	Score	*p*	Score	*p*	*p*
BSSI day 3—baseline	−9.44 ± 6.14	< 0.001	−5.40 ± 4.22	< 0.001	0.043
BSSI week 1—baseline	−10.68 ± 6.59	< 0.001	−7.47 ± 4.70	< 0.001	0.130
BSSI week 2—baseline	−13.75 ± 7.47	< 0.001	−10.33 ± 7.16	< 0.001	0.205
BSSI week 3—baseline	−14.50 ± 8.01	< 0.001	−10.00 ± 6.99	< 0.001	0.107
HDRS‐17 week 1—baseline	−7.50 ± 5.19	< 0.001	−9.33 ± 6.05	< 0.001	0.372
HDRS‐17 week 2—baseline	−12.88 ± 5.60	< 0.001	−12.40 ± 6.46	< 0.001	0.828
HDRS‐17 week 3—baseline	−13.81 ± 6.28	< 0.001	−13.27 ± 7.29	< 0.001	0.825
MADRS week 1—baseline	−10.56 ± 8.46	< 0.001	−11.47 ± 8.50	< 0.001	0.769
MADRS week 2—baseline	−18.13 ± 8.44	< 0.001	−16.40 ± 11.04	< 0.001	0.627
MADRS week 3—baseline	−20.00 ± 8.71	< 0.001	−17.20 ± 11.67	< 0.001	0.453

At the primary efficacy time point, the baseline to endpoint changes on the BSSI revealed a significant improvement in both active and sham tDCS groups compared with baseline (*p* < 0.001) (Table 2). Of note, active tDCS yielded a more promising treatment effect in BSSI decrease score compared with sham control (*p* < 0.05). This antisuicide effect tended to be sustained in the secondary endpoint in Week 1, Week 2, and Week 3 (*p* = 0.130, *p* = 0.205, *p* = 0.107; respectively). The simple mean effect of active tDCS in BSSI was superior to sham over time (*p* < 0.001).

Results for HDRS‐17 and MADRS represented a significant decrease in both active and sham tDCS groups (*p* < 0.001) at Week 1, Week 2, and Week 3, compared with baseline. No difference in the decrease of HDRS‐17 and MADRS was observed between the active and sham groups (*p* > 0.05). Such insignificance could also be observed in simple mean effect on HDRS‐17 and MADRS over time (*p* > 0.05).

### 3.3 Categoric Efficacy Outcome

**FIGURE 3:**
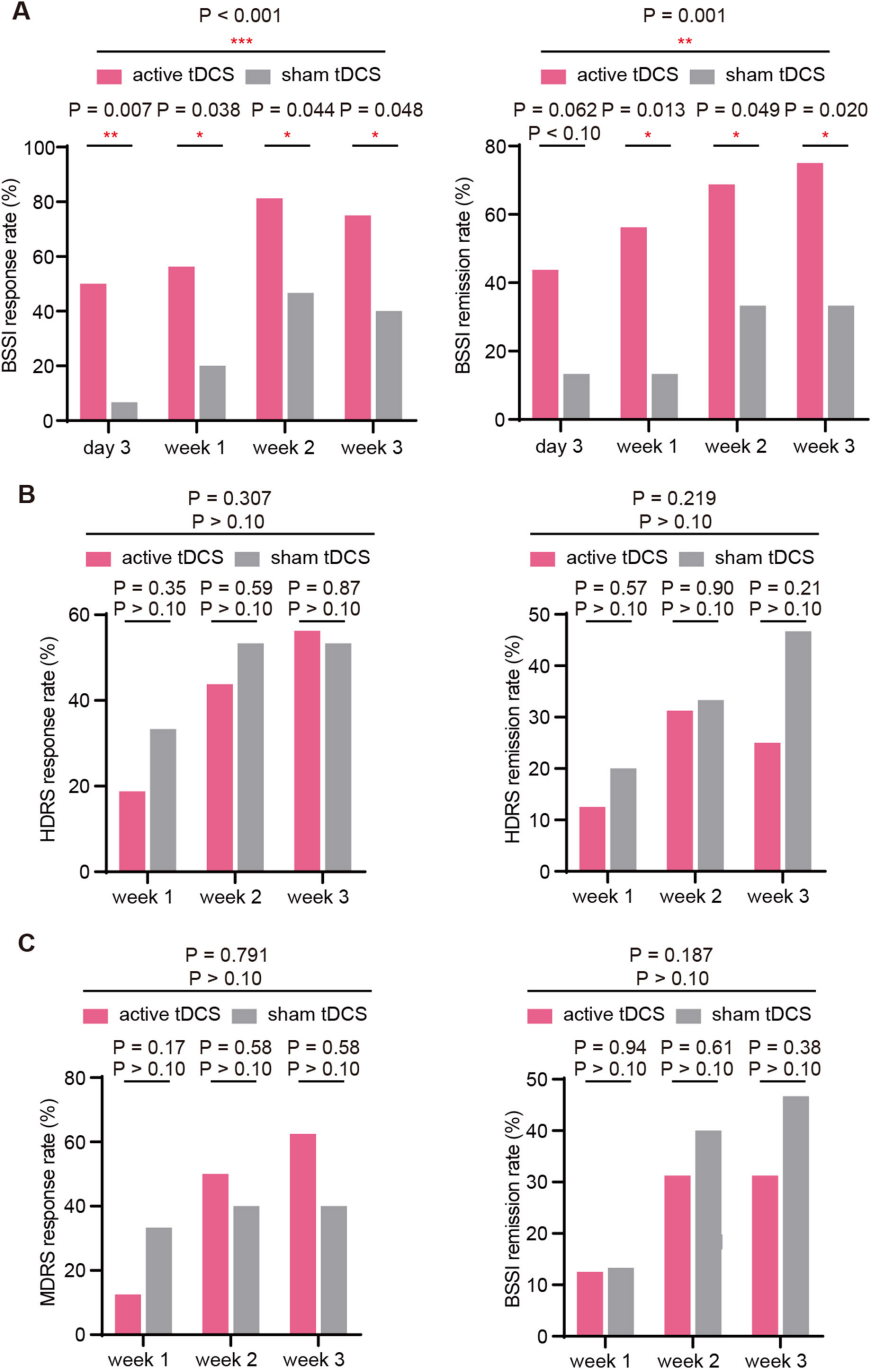
Response and remission rate in BSSI, HDRS‐17, and MDRS scores over time. (A–C) Response rate and remission rate in BSSI (A), HDRS‐17 (B), and MDRS (C) in each assessment point. With respect to response rate and remission rate, active tDCS was superior to sham in BSSI score, but not in HDRS‐17 and MDRS.

Efficacy results for categoric outcomes with the BSSI are shown in Figure 3.

Concerning tDCS ant‐suicide categoric response outcome, the response rate arrived as high as 50% on Day 3 under tDCS intervention, while sham control only showed a 6.7% response. Of note, it was elucidated that active tDCS revealed a higher response rate than the sham tDCS at Day 3 (*p* < 0.05), and this effect was sustained to Week 3 (*p* < 0.05). The simple mean effect was superior in active tDCS than sham control for BSSI response (*p* < 0.001) over time.

Similar results were also exemplified by the remission rate. The remission rate at Day 3 was as high as 43.8% in the active tDCS group, while the sham control only accounted for 13.3%. A higher remission rate in the active tDCS group, compared with the sham tDCS group, supported the efficacy of this treatment (Day 3: an increasing trend, *p* = 0.06; Week 1, Week 2, and Week 3: *p* < 0.05). The simple mean effect was superior in active tDCS than sham control for BSSI remission rate over time (*p* = 0.001).

Concerning the antidepressant categoric efficacy outcome, it was observed that there was no difference in response and remission rate between active and sham tDCS groups at Week 1, Week 2, and Week 3 (*p* > 0.05). The same results could also be found in the mean effect of response and remission rate (*p* > 0.05).

### 3.4 Safety Outcomes

For serious adverse effects, there were no deaths in this study, and no seizures were reported. Events reflecting disease‐related exacerbation, such as suicidality, mania switch, and exacerbation of depression, were not revealed (Table 3).

**TABLE 3:** Adverse reaction after active and sham tDCS intervention.

	Active tDCS (*n* = 16)		Sham tDCS (*n* = 15)		*p*
	*N*	Proportion	*N*	Proportion	
Tingling	6	37.5%	0	0	< 0.001
Death	0	0	0	0	—
Seizure	0	0	0	0	—
Suicidality	0	0	0	0	—
Mania switch	0	0	0	0	—
Exacerbation of depression	0	0	0	0	—

For spontaneous adverse effects, a total of six subjects (37.5%) reported adverse reactions to active tDCS (*p* < 0.001), while no adverse reactions were reported in the sham tDCS group. All of the patients with adverse reactions reported in the active group developed tingling pain on the scalp at the stimulation site, which mainly occurred during and after the treatment of active stimulation. The symptoms were mild and lasted only from a few minutes to a few hours. No patients dropped out of the trial due to adverse effects.

## 4 Discussion

In our first double‐blind, randomized, sham‐controlled research, based on the decrease score of BSSI, tDCS over left DLPFC yielded a rapid antisuicide effect at Day 3 in BD patients with suicidal ideation. This effect tended to remain in Week 1, Week 2, and Week 3, with only a trend revealed in continuous efficacy outcomes. However, when categoric efficacy outcome was examined, both response rate and remission rate showed significant improvement at almost every measure point. Surprisingly, the BSSI response rate arrived as high as 50% at Day 3 under tDCS intervention, while sham control only showed a 6.7% response. Also, the BSSI remission rate at Day 3 was as high as 43.8% in the active tDCS group, while the sham control only accounted for 13.3%. It is noteworthy that patients could respond to tDCS even after only 5 days of treatment [29], while in rTMS studies, trials that stimulated patients for longer periods achieved better results [30]. Intriguingly, our results demonstrate a shorter time window (i.e., 3 days) is effective and tolerable for patients. Altogether, the improvement in BSSI changes indicated the superiority of tDCS and quetiapine treatment, showing the great potential of tDCS for rapid antisuicide effect. Thus, our result first indicated the recommended evidence for tDCS to act as a rapid antidepressant treatment for bipolar depression patients with suicidal ideation.

It was well established that the activity of DLPFC decreases during the depression period and increases after antidepressant treatment [31]. However, the depression severity evaluated by HDRS‐17 and MADRS did not display a significant difference after the add‐on tDCS trial at Week 1, Week 2, and Week 3. Such insignificance could also be found in antidepressant categoric efficacy outcomes.

It is confirmed that tDCS is enable to enhance the excitatory synaptic transmissions through glutamate facilitation and GABA suppression in the cortex and modulate the neurotransmitter. Thus, tDCS may change the excitatory and inhibitory balance to achieve the treatment‐related neural events for BD patients with suicidal ideation [32, 33, 34]. Meanwhile, the synaptic plasticity and metabolic activity [19, 20, 21] possibly orchestrate the long‐term effect of symptom improvement. As reported, tDCS trials, similar to an add‐on treatment, were conducted on the DLPFC in BD patients with depression episodes, and it turned out that active tDCS with benzodiazepines showed significant efficacy in antidepressant effect than sham tDCS with benzodiazepines medication, in a small sample‐sized randomized clinical trial (active group, *n* = 30; sham group, *n* = 29) [35]. Likewise, a meta‐analysis consisting of 46 patients from 7 studies also suggested the antidepressant properties of tDCS treatment [36]. However, this pattern of effect was not consistent with our research, since our results did not show the antidepressant effect. Of note, one sham‐controlled experiment also detected no improvement in depression severity after tDCS treatment in 36 bipolar depression patients [37], hinting at the heterogeneity of tDCS treatment. The inconsistency in our results is possibly due to the heterogeneity of biased inclusion criteria of BD patients (i.e., whether the patients had suicidal ideation) and distinct experiment design (combination use with quetiapine). Namely, patients with prior suicidal attempts usually owned higher baseline HDRS depression scores [29]. Thus, the specificity in our antidepressant result of tDCS treatment might be due to the specific phenotype. Furthermore, it might also be due to the “ceiling effect” of the quetiapine medical treatment in dealing with depression episodes, as quetiapine was well‐recognized as the first‐line drug in treating bipolar depression [26]. Further single use of tDCS without medication or in combination with other drugs, brain stimulation therapy, or psychotherapy is required to confirm this effect.

Coincidentally, tDCS research with high quality also showed a contradictory antidepressant effect on unipolar patients [38, 39, 40], indicating that tDCS possibly has a subtle effect on neural modulation. Most tDCS studies in patients with BD were limited by their open‐label study design and/or small sample size. Thus, meta‐analysis and more sufficiently powered randomized controlled trials conducted on bigger sample sizes are awaiting the clarification of the effectiveness of tDCS in bipolar depression patients and even the BD subtype with suicidal ideation.

Taken together, it is of great controversy to elucidate the relationship between suicidal ideation and depression [41, 42]. Thus, whether we should treat suicidal ideation as treating depression or as an independent symptom is an elusive question [43]. Intriguingly, our result appeared to support the perspective that suicidal ideation and depression possibly were distinct, although associative, phenotypes, at least with respect to their discrepancy treatment effect of tDCS. Far more evidence is needed to identify this controversy.

However, it was promisingly supported that tDCS is an effective treatment across all bipolar disorder states. Not only for depression episodes, preliminary evidence demonstrated that tDCS improves neurological soft signs, cognition, and sleep quality in euthymia state [44, 45, 46]. Additionally, a case report also observed that tDCS as an add‐on to pharmacotherapy may improve manic symptoms [47]. These lines of evidence held the promise for clinical use of tDCS.

As for safety and tolerance concerns, those who received the active tDCS were significantly prone to have adverse reactions, all of which were tingling pain on the scalp. These adverse reactions were commonly reported after tDCS [22, 48] and were seemingly driven by the injected current in the scalp. However, these effects were usually mild and temporary and did not trigger death, seizure, or other serious adverse effects. No patients dropped out of the trial, validifying the tolerance of tDCS treatment. Of note, no mania switch was caused by tDCS, which further proved the safety of tDCS treatment. Meanwhile, according to the literature, limited cases had reported tDCS had the potential to induce hypomanic symptoms [44, 49, 50]. Most importantly, no reports of rapid cycling or increased risk of suicide associated with tDCS had been discovered, confirming the constricted side effect of tDCS treatment [36].

Altogether, we provided the first evidence that tDCS was effective as an antisuicide treatment for acute bipolar depression patients with suicidal ideation, with minimal side effects reported.

## 5 Limitations

To our knowledge, we pioneered applying tDCS as a suicidal intervention for bipolar patients with suicidal ideation. Although our study shed light on the rapid antisuicide effect of tDCS with an effective time window of at least 3 days, it existed some limitations that should be noted. First, the most important issue was the constricted sample size. Given that there were only 16 subjects in the active tDCS group and 15 subjects in the sham tDCS group, larger sample‐sized clinical trials can be carried out to further increase the credibility of the conclusion. Second, our clinical design did not include the long‐term effect of tDCS, as efficacy assessment ended at week 3 when tDCS treatment ceased. Future explorations can be conducted to investigate whether the treatment effect is persistent. Then, due to difficulties in sample collection, the present study did not single out the adolescent population for study but rather grouped it with adults into one large category for study, which may have negatively impacted the results. Additionally, the location of DLPFC in the present study was not individualized or MRI navigated, which may affect potentially the efficacy. Finally, sham tDCS in our study potentially represented a placebo effect [51, 52], which might concealed the true effect of active tDCS treatment. Thus, to better delineate the effects of tDCS, future clinical experiment designs could contain a waitlist control group to compare the active tDCS group.

## 6 Conclusion

In our first double‐blind, randomized, sham‐controlled research, based on the decreased score of BSSI, tDCS over left DLPFC yielded a rapid antisuicide effect at Day 3 in BD patients with suicidal ideation. This effect tended to remain in the 3‐week duration during tDCS treatment. Whereas, no superior antidepressant effect was revealed than the sham control. Thus, our result first indicated the recommended evidence for tDCS combined with quetiapine to act as a rapid antisuicide alternative treatment for bipolar depression patients, with minimal side effects reported.

## Author Contributions

**Dandan Wang:** conceptualization, methodology, formal analysis, data curation, writing – review and editing. **Xiaonan Guo:** formal analysis, data curation, writing – original draft, writing – review and editing. **Qi Huang**, **Zhong Wang**, and **Jingkai Chen:** methodology, data curation. **Shaohua Hu:** conceptualization, writing – review and editing, funding acquisition.

## Conflicts of Interest

The authors declare no conflicts of interest.

## Data Availability

All data in this study are available upon request by contact with the corresponding author.
